# The relationship between physical exercise and social anxiety among college students: the chain mediation of self-concept clarity and psychological resilience

**DOI:** 10.3389/fpsyg.2026.1886156

**Published:** 2026-08-12

**Authors:** Hongbo Zhao, Qingyang Chen

**Affiliations:** School of Physical Education, Liaoning Normal University, Dalian, China

**Keywords:** clarity of self-concept, college students, physical exercise, psychological resilience, social anxiety

## Abstract

**Background:**

With the widespread adoption of online social interactions, contemporary college students have shown a declining willingness to engage in face-to-face social interactions, and social anxiety has become an increasingly prominent issue, severely hindering the development of their interpersonal adaptation skills and their physical and mental well-being. Therefore, this study examines the relationship between physical exercise and social anxiety among college students and explores the chained mediating effects of self-concept clarity and psychological resilience on this relationship.

**Methods:**

Using a cross-sectional questionnaire survey design, a convenience sampling method was employed to survey 563 college students from universities in Liaoning, Henan, Hebei, Shandong, and Heilongjiang, using convenience sampling. Data were collected using the Physical Activity Level Scale, the Social Anxiety Scale, the Self-Concept Clarity Scale, and the Psychological Resilience Scale. SPSS 26.0 and AMOS 26.0 were used to perform descriptive statistics, Pearson correlation analysis, structural equation modeling, and Bootstrap mediation analysis.

**Results:**

(1) The results of the correlation analysis showed that the four variables—physical exercise, self-concept clarity, psychological resilience, and social anxiety—were significantly correlated with one another. Physical exercise was negatively correlated with social anxiety (*r* = −0.511), positively correlated with self-concept clarity (*r* = 0.545), and positively correlated with psychological resilience (*r* = 0.528). (2) The mediating effects of all three pathways were significant: Path 1 (physical exercise→self-concept clarity→social anxiety) had a mediating effect of −0.106, 95% CI [−0.168, −0.047]; Path 2 (physical exercise→psychological resilience→social anxiety) had a mediation effect of −0.139, 95% CI [−0.218, −0.085]; Path 3 (physical exercise→self-concept clarity→psychological resilience→social anxiety) had a chained mediation effect of −0.091, 95% CI [−0.139, −0.058].

**Conclusion:**

(1) Physical exercise is significantly negatively correlated with social anxiety among college students; (2) Self-concept clarity and psychological resilience mediate the relationship between physical exercise and social anxiety among college students in a chained manner; that is, physical exercise is associated with lower levels of social anxiety through both individual and chained mediating pathways.

## Introduction

The “Special Action Plan for Comprehensively Strengthening and Improving Student Mental Health Work in the New Era (2023–2025),” issued by China’s Ministry of Education and 16 other departments, explicitly states that physical exercise is recognized as having the potential to relieve stress and regulate emotions. It calls for promoting the deep integration of physical exercise with efforts to promote student mental health, thereby supporting students’mental health development (Central People’s Government of the People’s Republic of China, 2023). College is a critical period in early adulthood. After leaving the familiar social environments of home and high school, students begin to engage in interpersonal interactions independently. Therefore, this is a crucial stage in the development of social skills and psychological maturity for college students (Ross and Fuertes, 2010). According to the results of a survey on social anxiety among Chinese students, the prevalence of social anxiety symptoms among adolescents in elementary and secondary schools generally ranges from 17 to 22%, while the prevalence among college students reaches 22 to 30% (Tang et al., 2022). Social anxiety is a common psychological issue among college students and has become a significant factor affecting their mental health and personal development (Xie et al., 2023). Social anxiety refers to the emotional reactions of tension and fear, as well as avoidance behaviors, that individuals experience when in interpersonal situations or participating in social activities (Guo, 2000). Research indicates that the higher a college student’s level of social anxiety, the more likely they are to exhibit problems such as social withdrawal and communication difficulties (Üstündağ et al., 2025a; Üstündağ et al., 2025b). This not only makes it difficult to establish stable and harmonious peer relationships but also triggers negative emotions such as loneliness and depression, thereby adversely affecting their mental health (Ju et al., 2021). Severe social anxiety can also hinder the development of self-awareness, reduce an individual’s ability to adapt to the social environment, and even affect the healthy development of their personality (Alvi et al., 2020). Therefore, addressing social anxiety among college students is of great practical significance for maintaining their mental health and promoting their comprehensive development.

Previous studies have shown that physical exercise is significantly negatively correlated with social anxiety (Cheng et al., 2025); however, its underlying mechanisms—particularly the multivariate chain of transmission—have not yet been fully explored. Resource Conservation Theory posits that individuals have an innate tendency to accumulate multidimensional resources, including physiological, cognitive, and psychological resources, and that sufficient resource reserves can effectively cope with external stress and reduce levels of social anxiety (Rau, 2018). Physical exercise, as a positive form of physical and mental activity, is closely associated with an individual’s level of psychological resources (Moreno-Murcia et al., 2016). According to the self-enhancement hypothesis, physical exercise may be associated with more positive bodily experiences and clearer self-awareness, thereby exhibiting a statistical association with higher levels of self-concept clarity (Lindwall et al., 2014). Furthermore, higher levels of self-concept clarity may be associated with greater psychological resilience, which in turn may be associated with lower levels of social anxiety (Jia, 2022). Based on this, this study aims to construct a chained mediation model to thoroughly explore the relationship between physical exercise and social anxiety among college students, and to reveal the chained mediating effects of self-concept clarity and psychological resilience between the two variables, with the goal of providing theoretical support and practical guidance for universities seeking to alleviate social anxiety among college students through physical exercise interventions.

## Theoretical framework and hypotheses

### The relationship between physical exercise and social anxiety

Social anxiety is a common mental health issue among adolescents, characterized by individuals’ persistent experience of negative emotions—such as tension, fear, and worry—in social situations, as well as a tendency to avoid social activities (Beidel et al., 1995). Physical exercise refers to physical activities performed at a certain intensity, frequency, and duration, aimed at improving physical fitness and promoting overall health (Chi and He, 2008). Research indicates that physical exercise is significantly negatively correlated with social anxiety (Hui et al., 2025). The affect theory suggests that the emotional experiences generated during physical exercise can alleviate negative emotions such as anxiety and stress (Xu, 2023). As a typical form of positive physical activity, physical exercise stimulates the secretion of neurotransmitters during participation, directly inducing positive emotions such as pleasure, relaxation, and self-confidence. These positive emotions exert a certain antagonistic effect on social anxiety, diminishing an individual’s perception of tension in social situations and thereby reducing social anxiety (Chi and He, 2008; Hui et al., 2025). Therefore, this study proposes Hypothesis 1 (H1): Physical exercise is associated with a negative statistical prediction of social anxiety among college students.

### The mediating role of self-concept clarity

Self-concept clarity refers to how individuals perceive themselves and the extent to which they have a clear and well-defined sense of self (Campbell et al., 1996). Research indicates that physical exercise is significantly and positively correlated with self-concept clarity among college students (Yang, 2025). Physical exercise not only enhances individuals’ awareness and evaluation of their own bodies but also helps them form a clearer and more stable self-concept through sustained physical activity and firsthand personal experiences (Zhang et al., 2015). At the same time, students who regularly engage in physical exercise tend to have a clearer understanding of their own capabilities, thereby enhancing their self-concept clarity (Lee, 2021). Furthermore, In addition, the clarity of self-concept is significantly negatively correlated with levels of social anxiety (Jiang et al., 2025). According to the self-discrepancy theory, when an individual’s self-concept is unclear, they are prone to emotional distress, which exacerbates social anxiety (Yang and Chen, 2008). Individuals with higher self-concept clarity are able to navigate social interactions with greater confidence and exhibit lower levels of social anxiety (Quiñones and Kakabadse, 2015; Emery et al., 2015). Therefore, physical exercise may indirectly reduce social anxiety among college students by enhancing their self-concept clarity. Based on this, we propose Research Hypothesis 2 (H2) of this study: Self-concept clarity mediates the relationship between physical exercise and social anxiety among college students.

### The mediating role of psychological resilience

Psychological resilience is an inherent trait of an individual (Xi et al., 2015); it refers to the psychological capacity and process by which an individual copes, regulates, and adapts effectively when facing trauma, adversity, difficulties, and significant life stressors (Zhang, 2023). Research indicates a significant positive correlation between physical exercise and psychological resilience (Li Y. et al., 2024; Li D. B. et al., 2024; Li X. et al., 2024). On the one hand, physical exercise is associated with psychological resilience among college students (Li and Guo, 2023). Physical exercise can improve emotional and behavioral regulation by strengthening specific brain regions and large-scale neural circuits, thereby promoting an individual’s psychological resilience (Belcher et al., 2021). On the other hand, psychological resilience is a positive psychological resource that serves as a predictor of an individual’s physical and mental health (Wang, 2017). The psychological resilience model posits that psychological resilience, as a core psychological protective factor, can buffer the negative impact of stressful situations on an individual’s emotions and reduce the onset of anxiety (IJntema et al., 2023). Higher levels of psychological resilience can buffer the negative effects of social anxiety, enabling individuals to adopt more proactive approaches to resolving various social problems and to flexibly adjust their interpersonal relationships (Wang et al., 2012). Thus, psychological resilience, as an important protective factor, is closely related to social anxiety among college students. Based on this, we propose Research Hypothesis 3 (H3): Psychological resilience mediates the relationship between physical exercise and social anxiety among college students.

### Chain mediation between self-concept clarity and psychological resilience

Self-determination theory suggests that individuals with a well-developed self-concept are able to understand themselves accurately and rationally, and can consistently and promptly regulate their thoughts, emotions, and behaviors. When faced with difficulties, their coping strategies are typically positive, and they demonstrate strong psychological persistence and frustration tolerance (Ghorbani et al., 2010). Research indicates a significant positive correlation between self-concept clarity and psychological resilience (Jiang, 2014). Therefore, self-concept clarity may serve as a statistical predictor of psychological resilience levels and is one of the key factors in enhancing psychological resilience (Xi et al., 2011). Based on this, we propose Research Hypothesis 4 (H4): Self-concept clarity and psychological resilience may play a chained mediating role in the relationship between physical exercise and social anxiety among college students.

**Figure 1:**
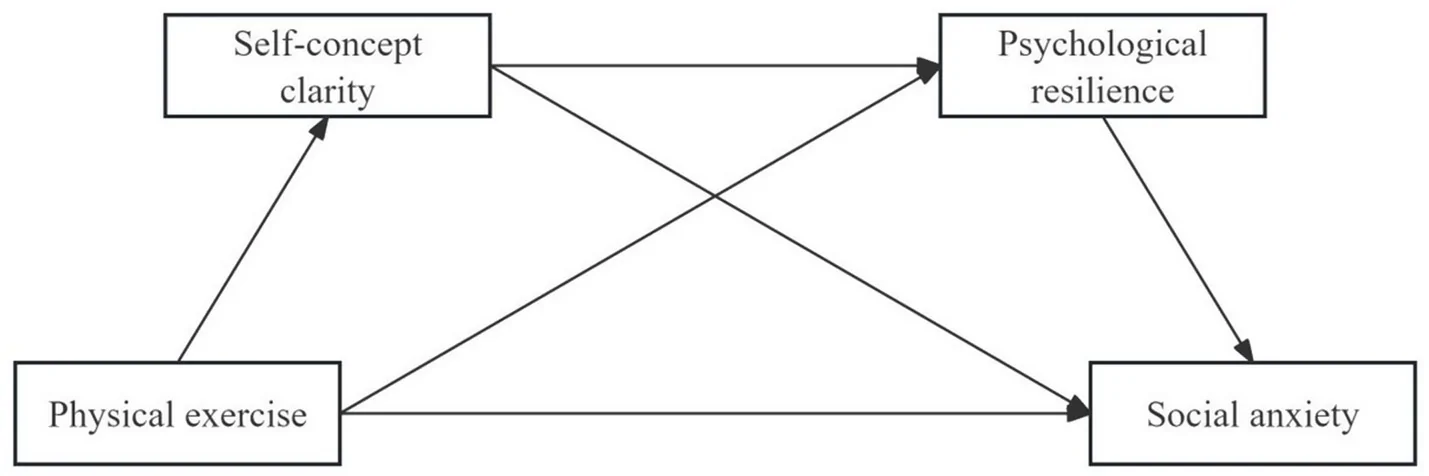
Hypothesis model.

Accordingly, the research hypothesis model is constructed as shown in Figure 1.

## Method

### Research design

This study employs a cross-sectional questionnaire survey design, with data collected simultaneously within a unified survey period. In terms of data analysis, independent-samples *t*-tests and one-way analysis of variance (ANOVA) were conducted sequentially to compare demographic differences; Pearson correlation analysis was used to preliminarily verify the relationships among variables; and finally, a chained mediation structural equation model was constructed to test the mediation effects. All survey activities for this study were conducted in 2026. The distribution of questionnaires, online responses, collection of raw data, and preliminary screening were all centralized between March 1, 2026, and April 30, 2026, with the overall survey period lasting a total of 2 months.

### Research participants

This study employed convenience sampling to select 563 college students from universities in five provinces—Liaoning, Henan, Hebei, Shandong, and Heilongjiang (including Dalian University of Technology, Liaoning University, Zhengzhou University, Yanshan University, Shenyang University, and Qingdao University of Science and Technology)—as survey participants. Within these institutions, the sample specifically covered the following major categories and distributions: 175 students (31.1%) in science and engineering, 240 students (42.6%) in humanities and history, and 148 students (26.3%) in arts and physical education. University counselors voluntarily forwarded the survey link to eligible students; participation was not mandated through group notifications or assigned tasks. The questionnaire covered five sections: basic information, level of physical activity, social anxiety, clarity of self-concept, and psychological resilience. No probability sampling procedures—such as random class assignment or random student selection—were employed throughout the process; students completed the questionnaire voluntarily and of their own accord.

Subject screening strictly adhered to uniform inclusion and exclusion criteria. Inclusion criteria: (1) Age between 18 and 25 years; (2) Currently enrolled in a full-time undergraduate program in China; (3) No history of hospital-diagnosed chronic physical or mental illnesses. Exclusion criteria: (1) Students in associate degree programs, graduate programs, or part-time continuing education programs; (2) Age under 18 or over 25 years; (3) Self-reported presence of chronic organic diseases or any diagnosed mental disorders, or those whose questionnaire responses did not meet quality standards. To accurately verify participant eligibility, the study employs a standardized information verification process. The basic information section of the questionnaire includes mandatory fields for date of birth, institution, program duration, and academic year to verify age and full-time undergraduate status. The questionnaire begins with a health screening multiple-choice question; if a participant has a diagnosed physical or mental illness, they are instructed to stop completing the questionnaire immediately, and the corresponding data will not be included in the analysis.

Regarding data confidentiality, a standardized informed consent module was included on the first page of the questionnaire, providing full disclosure of the study’s objectives, the fact that data would be used solely for academic analysis, and that all data would be processed anonymously throughout the study. This study was approved by the Ethics Committee of Liaoning Normal University (LL2026030), and all participants signed an informed consent form.

### Sample size estimation

A total of 606 questionnaires were distributed in this study, and all 606 were returned. The collected data were entered into an Excel spreadsheet, and invalid questionnaires (those completed in less than 2 min, containing repetitive responses, with numerous missing answers, or failing to meet age and health criteria) were excluded. Subsequently, SPSS 26.0 was used to perform statistical analysis on the collected data. Outliers were excluded based on the criterion that the absolute value of the Z-score for a given variable must be greater than 2, in order to minimize the impact of outliers on the statistical analysis results. Finally, a total of 43 invalid questionnaires were excluded, leaving 563 valid questionnaires, for a valid response rate of 92.90%. *A priori* power analysis was conducted using G*Power 3.1 software. The model included the independent variable (physical exercise), two mediators (self-concept clarity and psychological resilience), and three demographic control variables (gender, grade level, and major), for a total of six predictor variables; A moderate effect size of *f*^2^ = 0.15, a significance level of *α* = 0.05, and a statistical power of 1-*β* = 0.95 were set. Calculations indicated that the minimum sample size required to test for chain-mediated indirect associations was 146 cases. The valid sample of 563 cases in this study met the sample size requirement.

### Research tools

1. Physical activity scale

The Physical Activity Level Scale revised by Liang (1994) was used to assess college students’ physical activity levels. This scale consists of three dimensions—exercise intensity (e.g., “How intense is my physical activity?), exercise duration (e.g., “How long do I engage in physical activity at a time?), and exercise frequency (e.g., “How many times a month do I engage in physical activity?”)—each comprising one item. A 5-point Likert scale was used, with exercise intensity and frequency scored on a scale of 1–5, and duration scored on a scale of 0–4. The volume of physical exercise is equal to the product of exercise intensity, frequency, and duration. The total score on the scale ranges from 0 to 100, with higher scores indicating a greater volume of physical exercise. In this study, the Cronbach’s *α* coefficient for the Physical Activity Level Scale was 0.782. Although this scale’s Cronbach’s α was lower than that of the other scales used in this study, its reliability remains within an acceptable range (Lee and Comrey, 1978). Furthermore, previous studies have confirmed that this questionnaire has good construct validity (Ding et al., 2021).

1. Social anxiety scale

We used the Social Anxiety Scale developed by Leary (1983) based on his extensive clinical experience. The scale consists of 15 unidimensional items (e.g., “I feel nervous when I have to talk to a teacher”) and is scored using a 5-point Likert scale, where 1 corresponds to “strongly disagree” and 5 corresponds to “strongly agree.” The total score ranges from 15 to 75 points; a higher score indicates a higher level of social anxiety. The Chinese version of this scale has undergone standardization testing for a university student population (Li et al., 2024). In this study, the Cronbach’s *α* coefficient for this scale was 0.956.

1. Self-concept clarity scale

We used the scale developed by Campbell et al. (1996) and subsequently translated and revised by Niu et al. (2016). The questionnaire consists of 12 unidimensional items (e.g., “I spend a lot of time thinking about what kind of person I really am”) and employs a 5-point rating scale (1 = strongly disagree, 5 = strongly agree), including 9 reverse-scored items. The total score range for the scale is 12–60 points; a higher score indicates greater clarity of the individual’s self-concept. In this study, the Cronbach’s *α* coefficient for this scale was 0.926.

1. Psychological resilience scale

The Chinese version of the Connor-Davidson Resilience Scale (CD-RISC), translated and adapted by Yu and Zhang (2007), and others, was used to assess the level of psychological resilience among college students. The scale consists of 25 items, divided into the dimensions of tenacity, self-reliance, and optimism. The resilience dimension (e.g., “I can achieve my goals”) includes 13 items (items 11–23), while the self-reliance dimension (e.g.“I usually recover quickly after experiencing hardship or illness”) includes 8 items (items 1, 5, 7, 8, 9, 10, 24, 25), and Optimism (e.g., “I can cope with whatever happens”) includes 4 items (Items 2, 3, 4, 6). A 5-point scale is used, where 1 means “never” and 5 means “always.” The total score ranges from 25 to 125 points; a higher score indicates a higher level of psychological resilience. The Cronbach’s α coefficient for this scale is 0.941.

### Statistical analysis

Before conducting parametric tests, diagnostic checks for normality, linearity, and homogeneity of variance were performed on the data using SPSS 26.0. The results showed that the absolute values of skewness for all variables were less than 3 and the absolute values of kurtosis were less than 8, indicating good univariate normality and suitability for descriptive statistics and correlation analysis. Scatter plots showed that the variables exhibited a reasonable linear trend, and the *p*-values for Levene’s test of homogeneity of variance across all demographic subgroups were all greater than 0.05. Based on the fulfillment of the prerequisites for parametric tests, independent-samples t-tests and one-way ANOVA were used to compare differences in the variables among college students with different demographic characteristics, followed by descriptive statistics and Pearson correlation analysis. To examine the reliability and validity of the measurement model, confirmatory factor analysis was conducted using AMOS 26.0 on the Social Anxiety, Self-Concept Clarity, and Psychological Resilience scales. The measurement model’s convergent validity, discriminant validity, and overall model fit were comprehensively evaluated using standardized factor loadings, composite reliability (CR), average variance extracted (AVE), the Fornell-Larcker criterion, and model fit indices. To avoid the impact of high correlations among predictor variables on model estimates, this study conducted a multicollinearity diagnosis prior to regression analysis, using tolerance, the variance inflation factor (VIF), and the condition number as indicators to test for severe multicollinearity among the predictor variables. In this study, if the tolerance was greater than 0.20, the VIF was less than 5, and the condition index was less than 30, it was concluded that there was no significant multicollinearity, and regression analysis and structural equation modeling could proceed. However, since all variables in this study were collected via self-report questionnaires, the Harman single-factor test and the Unmeasured Latent Method Construct (ULMC) method were employed to examine common method bias. The Harman single-factor test was used for a preliminary assessment of common method bias, while the ULMC method was used to further verify whether common method bias had a substantial impact on model fit. Finally, a structural equation model (SEM) was constructed and the hypotheses were tested. Using AMOS 26.0, the model parameters were estimated via the maximum likelihood (ML) method. Based on the multivariate normality test, the critical kurtosis ratio for the observed variables in this study was 17.871, which is significantly greater than 1.96, indicating that the data did not meet the assumption of multivariate normality. Therefore, this study employed a bias-corrected bootstrap method with 5, 000 repetitions to correct for standard errors and mediating effect biases resulting from the non-normal distribution. A mediating effect was deemed significant if the 95% confidence interval did not include 0. This study set the significance level at *α* = 0.05 (two-tailed test) and reported the significance levels of the path coefficients based on the *p*-values (*p* < 0.05, *p* < 0.01, *p* < 0.001).

## Results

### Basic characteristics of the study participants

This study included a total of 563 valid university students. Among them, 302 were male (53.6%) and 261 were female (46.4%). The distribution by academic year was as follows: 117 first-year students (20.8%), 146 s-year students (25.9%), 135 third-year students (24.0%), and 165 fourth-year students (29.3%). The sample covered universities in five provinces—Liaoning, Henan, Hebei, Shandong, and Heilongjiang—and included students majoring in science and engineering (175, 31.10%), humanities and history (240, 42.60%), and arts and sports (148, 26.30%). The demographic characteristics of the study participants are shown in Table 1.

**Table 1:** Basic characteristics of the survey participants (*N* = 563).

Demographic variables	Category	Number of people	Percentage (%)
Gender	Male	302	53.60%
	Female	261	46.40%
Grade	Freshman year	117	20.80%
	Sophomore year	146	25.90%
	Junior year	135	24.00%
	Senior year	165	29.30%
Major	Humanities and history	240	42.60%
	Science and Engineering	175	31.10%
	Arts and athletics	148	26.30%

### Common method bias

Firstly, the Harman single-factor test was employed to rule out common method bias arising from the questionnaire method. The results of the factor analysis indicated that there were six factors with eigenvalues greater than 1; the first factor accounted for 33.96 per cent of the variance, which is well below the critical threshold of 40 per cent. It was therefore concluded that there was no significant common method bias in this study. To further rigorously control for potential bias, this study employed the Unidimensional Method Factor Control (ULMC) approach. Specifically, a latent common-method factor—capable of influencing all observed variables—was added to the original five-factor model. Model comparison results show that, following the inclusion of the method factor, the model’s fit indices were χ^2^/df = 1.331, RMSEA = 0.024 and CFI = 0.977. Compared with the baseline model (see Table 2), there was no significant improvement in any of the fit indices (ΔCFI = 0.001 < 0.01). This result indicates that, after controlling for methodological variation, the model structure and parameter estimates remain stable, suggesting that common method bias did not substantially interfere with the findings of this study.

**Table 2:** Model fit statistics.

Common indicators	χ2/df	GFI	RMSEA	CFI	NFI	TLI	AGFI	IFI
Criteria for assessment	<3	>0.9	<0.10	>0.9	>0.9	>0.9	>0.9	>0.9
Actual value	1.317	0.896	0.024	0.978	0.915	0.977	0.887	0.978

### Model validation

#### Aggregate validity testing

To examine the convergent validity and overall structural validity of the measurement model, this study conducted a confirmatory factor analysis using AMOS 26.0 on the three latent variables: social anxiety, self-concept clarity, and psychological resilience. The physical exercise variable, calculated as a total score (intensity × frequency × duration) using the Physical Activity Level Scale, is a unidimensional observed variable that does not meet the CFA requirement for multi-indicator measurement of latent variables; therefore, it was not included in the confirmatory factor analysis. The CFA results (Table 3) show that the standardized factor loadings for all observed items ranged from 0.663 to 0.826, all exceeding the critical threshold of 0.6, indicating that the items are well-representative of their latent variables. The composite reliability (CR) values for each latent variable ranged from 0.848 to 0.956, all exceeding the critical threshold of 0.7, indicating that the scale possesses good internal consistency. The average variance extracted (AVE) values ranged from 0.511 to 0.592, all exceeding the criterion of 0.5, indicating that the latent variables effectively explain more than half of the variance in their observed variables, demonstrating ideal convergent validity.

**Table 3:** CFA construct validity.

Latent variable	Manifest variable	Standard load coefficient	AVE	CR
Social anxiety	J1	0.801	0.592	0.956
	J2	0.813		
	J3	0.76		
	J4	0.783		
	J5	0.745		
	J6	0.767		
	J7	0.77		
	J8	0.726		
	J9	0.748		
	J10	0.819		
	J11	0.709		
	J12	0.776		
	J13	0.759		
	J14	0.738		
	J15	0.816		
Clarity of self-concept	Z1	0.737	0.511	0.926
	Z2	0.756		
	Z3	0.706		
	Z4	0.704		
	Z5	0.706		
	Z6	0.663		
	Z7	0.684		
	Z8	0.682		
	Z9	0.725		
	Z10	0.716		
	Z11	0.706		
	Z12	0.781		
Self-reliance	XZ1	0.761	0.53	0.9
	XZ2	0.748		
	XZ3	0.711		
	XZ4	0.733		
	XZ5	0.672		
	XZ6	0.706		
	XZ7	0.721		
	XZ8	0.769		
Optimism	XL1	0.788	0.583	0.848
	XL2	0.775		
	XL3	0.71		
	XL4	0.779		
Resilience	XJ1	0.822	0.587	0.949
	XJ2	0.826		
	XJ3	0.8		
	XJ4	0.741		
	XJ5	0.707		
	XJ6	0.764		
	XJ7	0.737		
	XJ8	0.777		
	XJ9	0.735		
	XJ10	0.739		
	XJ11	0.771		
	XJ12	0.76		
	XJ13	0.773		

#### Discriminant validity test

Discriminant validity was tested using the Fornell–Larcker criteria. As shown in Table 4, the values on the diagonal represent the square roots of the AVE for each latent variable, whilst the off-diagonal values represent the correlation coefficients between latent variables. The analysis results (Table 4) show that the square roots of the AVE for all latent variables (ranging from 0.715 to 0.769) are greater than the absolute values of the correlation coefficients between each latent variable and the others (the maximum absolute value being 0.583), indicating good discriminant validity among the latent variables.

**Table 4:** Results of the discriminant validity test (*n* = 563).

Constructs	Social Anxiety	Clarity of Self-Concept	Self-reliance	Optimism	Resilience
Social anxiety	0.769	−0.522	−0.393	−0.435	−0.503
Clarity of self-concept	−0.522	0.715	0.395	0.449	0.432
Self-reliance	−0.393	0.395	0.728	0.583	0.454
Optimism	−0.435	0.449	0.583	0.764	0.549
Resilience	−0.503	0.432	0.454	0.549	0.766

#### Measurement model fitting

As shown in Table 2, based on the evaluation criteria for model fit indices, most of the model fit indices—including CMINIDF, RMSEA, CFI, NFI, TLI and IFI—in the confirmatory factor analysis model used in this study meet the criteria. Although GFI (0.896) and AGFI (0.887) are slightly below 0.9, they are both above the acceptable threshold of 0.85 (Meydan and Şeşen, 2011). Taking into account the other excellent indicators, it is therefore concluded that the confirmatory factor model has good overall model fit.

#### Multicollinearity test

To test for multicollinearity among the predictor variables, a collinearity diagnosis was performed on the model. As shown in Table 5, the tolerance values for each predictor variable ranged from 0.610 to 0.672, all of which were greater than 0.20; the variance inflation factors (VIF) ranged from 1.489 to 1.640, all of which were less than 5; the maximum condition number is 10.074, which is less than 30. This indicates that there are no significant multicollinearity issues among the predictor variables, and regression analysis and structural equation modeling can proceed (See Table 5).

**Table 5:** Results of the multicollinearity test.

Metrics	Test Results	Criteria	Compliance
Tolerance	0.610 ~ 0.672	>0.20	Yes
Variance inflation factor (VIF)	1.489 ~ 1.640	<5	Yes
Maximum condition index	10.074	<30	Yes

### Descriptive statistics and correlation analysis

An independent samples *t*-test was used to examine gender differences across the various study variables. The results (see Table 6) show that, on the physical exercise dimension, the scores for male students (40.48 ± 25.55) were significantly higher than those for female students (34.24 ± 23.47), *p* = 0.003, Cohen’s *d* = 0.25. Although this difference reached statistical significance, the effect size was small (0.2 ≤ Cohen’s *d* < 0.5 is considered a small effect; 0.5 ≤ Cohen’s *d* < 0.8 is a medium effect; Cohen’s *d* ≥ 0.8 indicates a large effect), suggesting that gender differences are only practically significant in relation to physical exercise. In the three dimensions of social anxiety (*p* = 0.298, *d* = 0.09), self-concept clarity (*p* = 0.431, *d* = 0.07) and psychological resilience (*p* = 0.551, *d* = 0.05), gender differences did not reach statistical significance (*p* > 0.05), and the effect sizes were all well below 0.2. This suggests that these variables are expressed in a largely consistent manner among male and female university students, with no practical gender differences present.

**Table 6:** Gender differences across various variables (N = 563).

Variables	Gender	Number of people	M ± SD	*t*	df	*p*	Cohen’s *d*
Physical exercise	Male	302	40.48 ± 25.55	3.00	561	0.003	0.25
	Female	261	34.24 ± 23.47				
Social anxiety	Male	302	42.05 ± 16.58	1.04	561	0.298	0.09
	Female	261	40.59 ± 16.51				
Clarity of self-concept	Male	302	37.31 ± 11.18	0.79	561	0.431	0.07
	Female	261	36.55 ± 11.54				
Psychological resilience	Male	302	59.25 ± 20.76	0.60	561	0.551	0.05
	Female	261	58.20 ± 21.32				

*p < 0.05, **p < 0.01, ***p < 0.001.

Using one-way analysis of variance (ANOVA), this study compared differences among college students in different academic years in terms of physical exercise, social anxiety, self-concept clarity, and psychological resilience. The results indicate that there were significant differences among college students in different academic years in physical exercise (*F* = 0.654, *p* = 0.581, ηp^2^ = 0.005), social anxiety (*F* = 0.990, *p* = 0.397, ηp^2^ = 0.005), self-concept clarity (*F* = 0.497, *p* = 0.685, ηp^2^ = 0.003), and psychological resilience (*F* = 1.377, *p* = 0.249, ηp^2^ = 0.007) did not reach statistical significance (*p* > 0.05), and the effect sizes were all small (ηp^2^ < 0.01), indicating that the actual influence of grade level on these variables was weak and that *post hoc* multiple comparisons were not necessary (See Table 7).

**Table 7:** Grade-level differences across various variables (*n* = 563).

Variables	Grade	Number of people	M ± SD	*F*	*P*	ηp^2^
Physical exercise	Freshman year	117	36.45 ± 25.05	0.654	0.581	0.005
	Sophomore year	146	38.67 ± 23.54			
	Junior year	135	35.61 ± 25.52			
	Senior year	165	39.05 ± 25.11			
Social anxiety	Freshman year	117	42.95 ± 15.80	0.990	0.397	0.005
	Sophomore year	146	40.17 ± 15.90			
	Junior year	135	42.47 ± 17.22			
	Senior year	165	40.42 ± 17.07			
Clarity of self-concept	Freshman year	117	36.10 ± 11.11	0.497	0.685	0.003
	Sophomore year	146	37.75 ± 11.79			
	Junior year	135	37.13 ± 11.00			
	Senior year	165	36.73 ± 11.44			
Psychological resilience	Freshman year	117	55.29 ± 21.61	1.377	0.249	0.007
	Sophomore year	146	60.09 ± 20.46			
	Junior year	135	59.53 ± 21.82			
	Senior year	165	59.42 ± 20.31			

*p < 0.05, **p < 0.01, ***p < 0.001.

A one-way analysis of variance (ANOVA) was conducted to compare differences among college students from different majors in terms of physical exercise, social anxiety, self-concept clarity, and psychological resilience. The results (see Table 8) showed that social anxiety (*F* = 1.164, *p* = 0.313, ηp^2^ = 0.004), self-concept clarity (*F* = 0.647, *p* = 0.524, ηp^2^ = 0.002), and psychological resilience (*F* = 0.316, *p* = 0.729, ηp^2^ = 0.001) did not reach statistical significance across different majors (all p > 0.05), and the effect sizes were all small (ηp^2^ < 0.01). Differences among students from different majors were statistically significant only for physical exercise (*F* = 7.336, *p* = 0.001, ηp^2^ = 0.026), with a small effect size, indicating that major type has a certain influence on college students’ levels of physical exercise. Further post hoc comparisons using the LSD method (see Table 9) revealed that students in arts and physical education majors scored significantly higher on physical exercise than those in humanities and social sciences majors (*p* < 0.001) and science and engineering majors (*p* = 0.033). The difference between humanities and social sciences majors and science and engineering majors was not statistically significant (*p* = 0.103).

**Table 8:** Differences in specializations across variables (*n* = 563).

Variables	Major	Number of Students	M ± SD	*F*	*P*	ηp^2^
Physical exercise	Humanities and History	240	33.78 ± 22.97	7.336	0.001	0.026
	Science and Engineering	175	37.75 ± 25.25			
	Arts and Athletics	148	43.58 ± 25.98			
Social anxiety	Humanities and History	240	41.84 ± 16.14	1.164	0.313	0.004
	Science and Engineering	175	42.23 ± 16.26			
	Arts and Athletics	148	39.61 ± 17.52			
Clarity of self-concept	Humanities and History	240	36.49 ± 10.88	0.647	0.524	0.002
	Science and Engineering	175	36.86 ± 11.95			
	Arts and Athletics	148	37.83 ± 11.39			
Psychological resilience	Humanities and History	240	58.10 ± 21.05	0.316	0.729	0.001
	Science and Engineering	175	58.77 ± 21.95			
	Arts and Athletics	148	59.84 ± 19.88			

*p < 0.05, **p < 0.01, ***p < 0.001.

**Table 9:** *Post-hoc* comparison (LSD) of physical activity levels among college students in different majors.

Comparison groups	Mean difference (I–J)	Standard error	95% CI	*p*
Humanities and liberal arts–science and engineering	−3.974	2.436	[−8.76, 0.81]	0.103
Humanities and liberal arts–arts and athletics	−9.806	2.561	[−14.84, –4.78]	<0.001
Science and engineering–arts and athletics	−5.833	2.736	[−11.21, –0.46]	0.033

*p < 0.05, **p < 0.01, ***p < 0.001.

The results of the correlation analysis show (Table 10) that physical exercise was significantly negatively correlated with social anxiety (*r* = −0.511, *p* < 0.01) and significantly positively correlated with both self-concept clarity and psychological resilience (*r* = 0.545, 0.528, *p* < 0.01). Social anxiety was significantly negatively correlated with both self-concept clarity and psychological resilience (*r* = −0.491, −0.523; *p* < 0.01). Self-concept clarity was significantly positively correlated with psychological resilience (*r* = 0.475, *p* < 0.01). Regarding demographic variables, gender was significantly negatively correlated with physical exercise (*r* = −0.126, *p* < 0.01), but showed no significant correlations with social anxiety, self-concept clarity, or psychological resilience. Grade level was not significantly correlated with any of the main variables (physical exercise, social anxiety, self-concept clarity, or psychological resilience). Major was significantly positively correlated with physical exercise (*r* = 0.159, *p* < 0.01), but showed no significant correlations with social anxiety, self-concept clarity, or psychological resilience.

**Table 10:** Correlation analysis of physical exercise, social anxiety, self-concept clarity, and psychological resilience among college students.

Variable	1	2	3	4	5	6	7
Gender (1)	1						
Grade (2)	−0.075	1					
Major (3)	−0.080	0.017	1				
Physical activity (4)	−0.126**	0.023	0.159**	1			
Social anxiety (5)	−0.044	−0.034	−0.049	−0.511**	1		
Self-concept clarity (6)	−0.033	0.007	0.046	0.545**	−0.491**	1	
Psychological resilience (7)	−0.025	0.055	0.033	0.528**	−0.523**	0.475**	1

*p < 0.05, **p < 0.01, ***p < 0.001.

### Construction of the structural equation model

In this study, the model was constructed using AMOS 26.0, and the Maximum Likelihood (ML) estimation method was employed for parameter estimation. The independent variable, physical exercise (X), was included in the model as an observed variable based on a composite total score calculated as the product of intensity, frequency, and duration; The mediating variable, self-concept clarity (M1), and the dependent variable, social anxiety (Y), are first-order latent variables, both measured by mature unidimensional scales; the full 12-item and 15-item versions of the scales, respectively, were used as observed indicators. The mediating variable, psychological resilience (M2), was defined as a second-order latent variable comprising the three dimensions of optimism, self-reliance, and tenacity, and these dimensions were used as observed indicators. In the model, gender and major are set as manifest control variables. To simplify the model, only social anxiety is directly predicted. No controls are applied to the independent or mediating variables.

As shown in Table 11, χ^2^/df = 1.576 < 3, indicating that the model fit is acceptable. GFI = 0.917, NFI = 0.93, IFI = 0.973, TLI = 0.971, and CFI = 0.973—all greater than 0.9—indicate that the model has good fit validity, while RMSEA = 0.032 < 0.08 indicates an acceptable data fit. In summary, this measurement model meets the ideal standards and can be used to further test the mediating effects.

**Table 11:** Model fit.

Fitting metrics	Criteria	Actual values	Fitting results
Absolute fit indices			
GFI	>0.9	0.917	Good
AGFI	>0.9	0.905	Good
RMSEA	<0.08	0.032	Good
Value-added fit indices			
NFI	>0.9	0.930	Good
IFI	>0.9	0.973	Good
TLI	>0.9	0.971	Good
CFI	>0.9	0.973	Good
Simplified fit indices			
CMIN/DF	<3	1.576	Good
PNFI	>0.5	0.863	Good
PGFI	>0.5	0.801	Good

**Figure 2:**
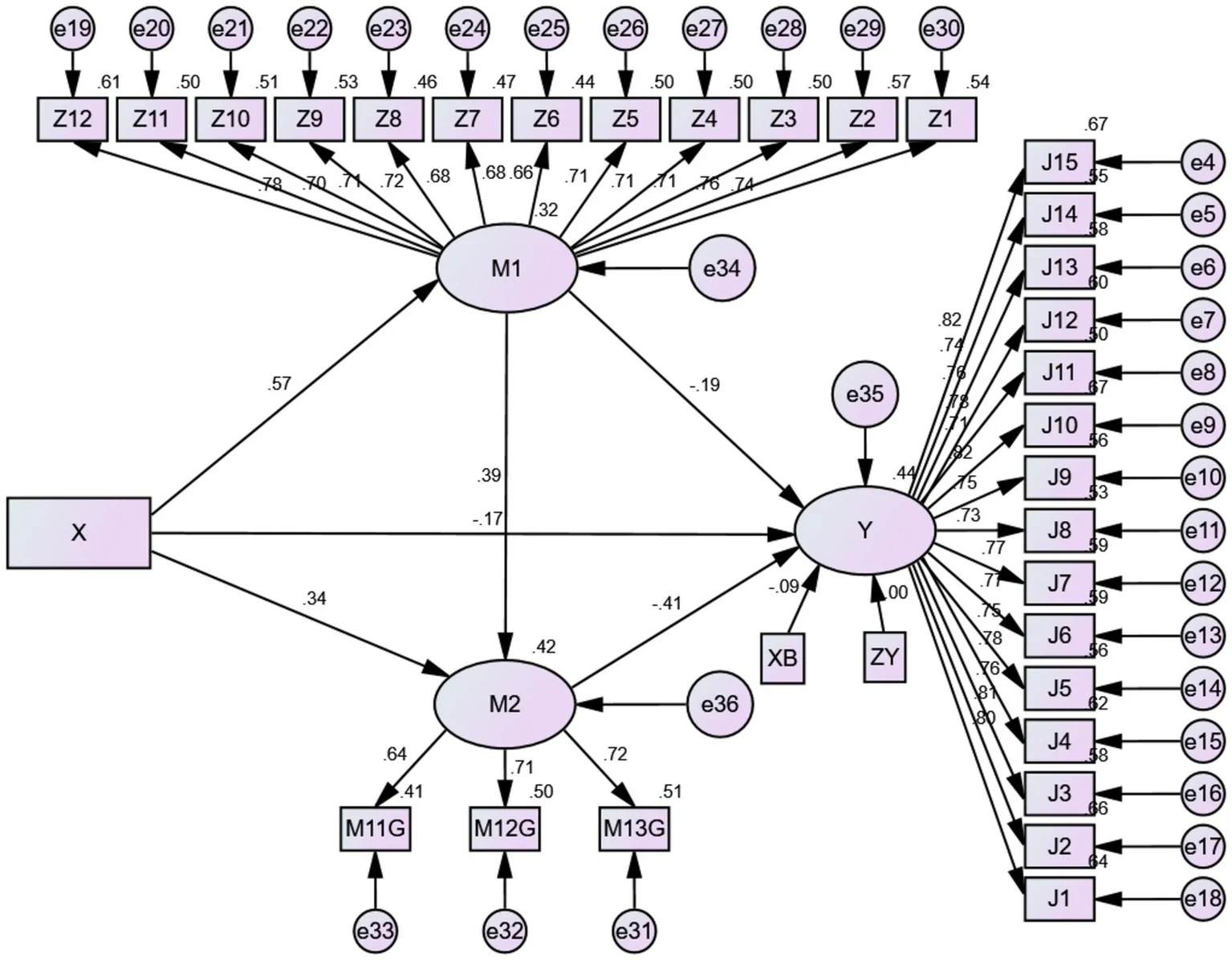
SEM mediating model analysis diagram.

As shown in the SEM mediation model analysis figure and Table 12, the path effects among all core variables were statistically significant (*p* < 0.001). Specifically, physical exercise was significantly and positively associated with self-concept clarity (*β* = 0.568, *p* < 0.001) and psychological resilience (*β* = 0.392, *p* < 0.001), while it was significantly and negatively associated with social anxiety (*β* = −0.171, *p* < 0.001). Self-concept clarity was significantly positively associated with psychological resilience (*β* = 0.34, *p* < 0.001) and significantly negatively associated with social anxiety (*β* = −0.186, *p* < 0.001). Psychological resilience was significantly negatively associated with social anxiety (*β* = −0.409, *p* < 0.001). The model’s explained variance for the endogenous variables was 0.323 for self-concept clarity, 0.42 for psychological resilience, and 0.443 for social anxiety. This indicates that the model used in this study has strong explanatory power (Figure 2).

**Table 12:** Path analysis of the structural equation model.

Path	Standard path coefficient	Non-standard path coefficient	S.E.	C.R.	*P*	*R* ^2^
Physical exercise → self-concept clarity	0.568	0.599	0.045	13.309	***	0.323
Physical exercise → psychological resilience	0.392	3.923	0.574	6.836	***	0.42
Self-concept clarity → psychological resilience	0.34	3.585	0.558	6.423	***	
Self-concept clarity → social anxiety	−0.186	−0.228	0.063	−3.613	***	
Psychological resilience→ social anxiety	−0.409	−0.05	0.008	−6.415	***	
Physical exercise → social anxiety	−0.171	−0.22	0.061	−3.625	***	0.443
Gender → social anxiety	−0.091	−0.21	0.078	−2.692	0.007	
Major → social anxiety	0.002	0.003	0.048	0.055	0.956	

*p < 0.05, **p < 0.01, ***p < 0.001.

We used a nonparametric percentile bootstrap method with bias correction (5, 000 repetitions) to test the significance of the mediating effect. As shown in Table 13, the estimated value of the total effect is −0.507, with a 95% confidence interval of [−0.577, −0.426], which does not include 0, indicating that the overall negative effect of the independent variable (physical exercise) on the dependent variable (social anxiety) is significant. The direct effect of physical exercise on social anxiety was −0.171, with a 95% confidence interval of [−0.266, −0.063], which also does not include 0, indicating that the direct effect is significant and accounts for 33.73% of the total effect.

**Table 13:** Analysis of mediating effects.

Effect	Parameter	Estimate	Lower	Upper	P	Percentage of the Effect
Direct effect	Physical exercise → social anxiety	−0.171	−0.266	−0.063	0.003	33.73%
Indirect effect	Physical exercise → self-concept clarity → social anxiety	−0.106	−0.168	−0.047	0.001	20.91%
	Physical exercise → psychological resilience → social anxiety	−0.139	−0.218	−0.085	0.001	27.42%
	Physical exercise → self-concept clarity → psychological resilience → social anxiety	−0.091	−0.139	−0.058	0.001	17.95%
Total effect	Physical exercise → social anxiety	−0.507	−0.577	−0.426	0.001	-

*p < 0.05, **p < 0.01, ***p < 0.001.

Regarding the indirect effect tests, the Bootstrap confidence intervals for all three mediating paths did not include 0, indicating that the mediating effects are significant. Specifically: (1) The indirect effect value for the path“physical exercise→self-concept clarity→social anxiety”is −0.106, accounting for 20.91% of the total effect; (2) The indirect effect value for the path “Physical Exercise → Psychological Resilience→Social Anxiety” was −0.139, accounting for 27.42% of the total effect; (3) The indirect effect value for the chained mediation path“Physical Exercise→Self-Concept Clarity→Psychological Resilience→Social Anxiety”was −0.091, accounting for 17.95% of the total effect.

## Discussion

### The relationship between physical exercise and social anxiety

First, existing research has largely examined the mechanisms underlying the relationship between physical exercise and social anxiety through a single mediating variable or a specific chain of pathways. For example, Wang and Chen (2023), using a sample of general college students, revealed that physical exercise is associated with social anxiety through the chain of mediation “body image → self-esteem” (Wang and Chen, 2023). Second, in their study on college students’ physical activity participation, Yang et al. (2026) introduced external environmental factors and revealed a chained association between “family support and self-efficacy” and the relationship between physical activity participation and mental health (Yang et al., 2026). Finally, in a comparative study of chained mediation involving mindfulness and psychological resilience—which also used a sample of Chinese college students—the researchers found that physical exercise may be associated with social anxiety through the “mindfulness →psychological resilience” chained pathway (Jiang et al., 2025). In summary, previous studies have generally focused on exploring various indirect mediation pathways, while paying relatively little attention to the direct relationship between physical exercise and social anxiety (Xiao et al., 2025).

Based on existing research, physical exercise may be associated with changes in the levels of neurotransmitters such as endorphins and dopamine, as well as a reduction in stress hormones like cortisol, and is thus linked to improvements in negative emotions such as anxiety and tension (Anderson and Shivakumar, 2013). On the other hand, physical exercise provides college students with opportunities to interact with others (Li and Zizzi, 2018). Particularly in team sports, individuals can establish positive interpersonal relationships through cooperation and communication, which helps foster positive social connections and may be associated with an enhanced sense of social belonging and a reduced tendency toward social avoidance (Fu, 2025). Taken together, the results of this study suggest that physical exercise is significantly associated with social anxiety among college students. This association may involve mechanisms across multiple levels—including physiological, psychological, and social interaction—and provides empirical evidence to support the implementation of physical education programs in higher education institutions to promote students’ mental health.

### The mediating role of self-concept clarity

Self-concept clarity refers to the degree of certainty and internal consistency in an individual’s self-perception and is regarded as an important cognitive foundation of mental health (Campbell et al., 1996). This study shows that self-concept clarity plays a significant mediating role between physical exercise and social anxiety among college students. Although previous studies have confirmed a negative association between self-concept clarity and social anxiety (Liu et al., 2017), and physical exercise has been shown to enhance “bodily self-concept” (Zhang et al., 2015), most of these findings have either focused on self-evaluation at the bodily level or examined only the direct effects of self-concept clarity. Consequently, they have failed to fully elucidate the dynamic process through which physical exercise is systematically linked to an individual’s cognitive clarity regarding their overall self—and, by extension, to their levels of social anxiety. The unique contribution of this study lies in expanding the concept of self-concept clarity from the physical self-concept to the level of overall self-concept, clearly demonstrating that physical exercise is associated with a relatively stable relationship with overall self-concept clarity among college students. Specifically, on the one hand, as individuals engage in physical exercise, they may gain a clearer understanding of their own abilities and personality traits by achieving exercise goals and overcoming personal challenge. This process may be associated with higher levels of self-concept clarity (Sun et al., 2014). When individuals have a clearer understanding of themselves, they are less likely to experience self-doubt or anxiety in social situations due to others’ evaluations, which is associated with lower levels of social anxiety (Chen L. T. et al., 2025; Chen X. et al., 2025). On the other hand, college students with a clear self-concept are able to view their own strengths and weaknesses objectively. They do not rely on others’ negative evaluations to define themselves, nor do they excessively fear rejection by others in social situations, thereby reducing the occurrence of social anxiety (Zhang et al., 2019). In contrast, college students with an unclear self-concept are prone to falling into a state of self-denial and overanalyzing others’ thoughts, making them more likely to exhibit higher levels of social anxiety (Li and Xu, 2022).

### The mediating role of psychological resilience

First, the findings of this study are consistent with the conclusions of a large-scale survey by Li et al. (2024) involving 530 Chinese college students. That study found that physical exercise was negatively correlated with symptoms of social anxiety and positively correlated with psychological resilience, and that physical exercise may be associated with lower levels of social anxiety through an independent mediating pathway involving psychological resilience (Wang Z. et al., 2024; Wang Z. F. et al., 2024). Second, Wang Z. et al. (2024) and Wang Z. F. et al. (2024) also noted in a moderated mediation model that moderate physical exercise can effectively reduce negative emotions such as anxiety and depression, a finding that may be attributed to the association between physical exercise and higher levels of psychological resilience (Zhu and Liu, 2021). Furthermore, Zhu and Liu (2021), in a study involving 346 college students with hearing impairments, found that physical exercise indirectly influences social anxiety through the mediating effect of psychological resilience, supporting the mediating role of psychological resilience in the relationship between the two (Cui et al., 2023). Finally, a post-pandemic study by Cui et al. (2023) indicated that psychological resilience plays a significant mediating role in the relationship between physical exercise and anxiety, confirming that psychological resilience is a core psychological buffer variable through which physical exercise improves emotional health (Chen L. T. et al., 2025; Chen X. et al., 2025).

This study deepens our understanding of this concept. Specifically, physical exercise itself possesses certain challenging and stress-coping attributes (Wang Z. et al., 2024; Wang Z. F. et al., 2024). As college students engage in exercise, they must continually overcome physical challenges and persevere to achieve their fitness goals; the exercise experiences accumulated during this process may be associated with higher levels of stress resilience and psychological resilience (Wang Z. et al., 2024; Wang Z. F. et al., 2024). Psychological resilience serves as a vital psychological resource for individuals in coping with stress, setbacks, and other negative situations (Wang and bin Shuib, 2023). When college students face social anxiety, those with high psychological resilience are often able to quickly regulate negative emotions and actively cope with social challenges (Chen L. T. et al., 2025; Chen X. et al., 2025). This study further suggests that psychological resilience may serve as a key psychological mediating mechanism between physical exercise and social anxiety, highlighting the unique value of physical exercise in fostering psychological resilience among college students. It also suggests that universities can provide students with more opportunities to participate in physical activities—such as through regular exercise programs and organized team sports competitions—to promote their mental health.

### The chain mediation effect of self-concept clarity and psychological resilience

Research on physical exercise and self-concept clarity has provided preliminary evidence of a positive association between the two. Physical exercise is considered an important behavioral pattern closely related to an individual’s self-perception and has been shown to be significantly positively correlated with college students’ levels of self-concept clarity. Jiang (2014) revised the Adolescent Self-Concept Clarity Scale and used multiple regression analysis to examine the distinct effects of self-concept clarity and self-esteem on psychological resilience, finding that both self-concept clarity and self-esteem are associated with psychological resilience (Jiang, 2014). Although previous studies have examined the mediating effects of self-concept clarity and psychological resilience separately, or explored their chained mediating roles in the relationships between these two variables and other factors, few studies have integrated them in a sequential manner into a comprehensive explanatory framework of “physical exercise → social anxiety.” Luo et al. (2025) found that among endoscopy nurse specialists, psychological resilience and self-concept clarity exerted a chained mediating effect between perceived job benefits and job burnout, indicating that the chained combination of self-concept clarity and psychological resilience is applicable across different populations when explaining various psychological phenomena.

The results of this study suggest that individuals with higher self-concept clarity may possess a more stable foundation of self-awareness, making it easier for them to identify and mobilize their psychological resources when facing stressful situations, and that this is associated with higher levels of psychological resilience (Cai et al., 2018). Furthermore, higher psychological resilience may help individuals maintain emotional stability and adopt more positive coping strategies in social situations, and is associated with lower levels of social anxiety (Nie et al., 2011). This study found that self-concept clarity and psychological resilience form a chain-like association between physical exercise and social anxiety, further enriching the explanatory pathways for the relationship between physical exercise and social anxiety among college students.

### Limitations

Given the cross-sectional study design, it is not possible to infer causal relationships among the variables. Although structural equation modeling was used to analyze the path relationships among the variables, it was not possible to strictly establish the causal sequence between them. For example, while it is theoretically possible that physical exercise enhances psychological resilience, it cannot be ruled out that individuals with lower levels of social anxiety are inherently more inclined to engage in physical exercise. Second, the sample was selected using convenience sampling, resulting in a lack of representativeness. This study employed convenience sampling, with the sample primarily concentrated in select universities across five provinces—Liaoning, Henan, Hebei, Shandong, and Heilongjiang—and the sample size was unevenly distributed across different regions and types of institutions. This sampling method may lead to sampling bias, making it difficult to generalize the study’s conclusions to college student populations in other parts of the country with different cultural backgrounds or educational environments. Third, the single source of data and potential biases. All variable data in this study relied on self-reports from the participants. On the one hand, self-report methods are susceptible to social desirability, memory biases, and participants’ current emotional states, which may lead to subjective biases in the data; on the other hand, although this study statistically controlled for common-method bias using Harman’s one-factor test and the Unbiased Method of Control (ULMC), data from a single source still carries a potential risk of measurement error. Fourth, the lack of objectivity in measuring physical exercise.

This study used the Physical Activity Scale for subjective self-assessment and lacked objective physical measurement devices (such as accelerometers or heart rate monitoring wristbands) to quantitatively monitor the actual physical activity levels of college students. Subjectively reported exercise intensity, frequency, and duration may differ from the actual physical exertion, thereby affecting the measurement accuracy of the independent variables.

### Future prospects

First, longitudinal tracking and intervention studies will be conducted to further validate the causal relationships among the variables. This study employed a cross-sectional design, which only revealed the associations among physical exercise, social anxiety, self-concept clarity, and psychological resilience; it cannot yet clearly establish the causal relationships among these variables. Future research could adopt a longitudinal design to measure physical exercise, self-concept clarity, psychological resilience, and social anxiety among college students at multiple time points, using methods such as cross-lagged models to further examine the dynamic relationships among these variables. Additionally, randomized controlled intervention studies could be conducted, using physical exercise as the intervention, to further validate the mechanisms through which physical exercise alleviates social anxiety and its long-term effects. Second, the scope of the study population and sample size could be expanded to test the stability and generalizability of the findings. The sample in this study was primarily drawn from universities in Liaoning, Henan, Hebei, Shandong, and Heilongjiang, which limits the representativeness of the sample. Future research could employ methods such as stratified random sampling to expand the sample pool, covering college students from different regions, educational levels, and academic backgrounds. It should also further examine whether the chain mediation model varies across different genders, grade levels, majors, and cultural backgrounds, thereby enhancing the external validity and practical applicability of the research findings. Third, research methods and measurement approaches should be diversified to thoroughly elucidate the mechanisms through which physical exercise influences social anxiety. Future studies could integrate multi-source data—including subjective questionnaires, self-reports, and peer evaluations—while incorporating objective measurement devices such as fitness trackers and accelerometers to enhance the accuracy of physical exercise measurements. At the same time, by integrating physiological indicators (such as heart rate variability and cortisol levels) and neurocognitive indicators, future research can further explore the mechanisms through which physical exercise influences social anxiety from behavioral, physiological, and psychological perspectives, thereby providing more robust empirical evidence to support mental health promotion and physical activity interventions in higher education institutions.

### Innovative contributions and practical implications

This study is the first to integrate self-concept clarity and psychological resilience into a chained mediation model, overcoming the limitations of previous research that often focused on a single mediating variable. The findings not only validate the relationship between physical exercise and social anxiety but also clearly reveal a dual psychological transmission pathway from “cognitive resources (self-concept clarity)” to “coping resources (psychological resilience).” This enriches the empirical evidence supporting the application of Resource Conservation Theory in the field of sports psychology and provides new empirical grounds for understanding the complex mechanisms through which physical exercise promotes mental health.

In practice, the positive effects of physical exercise on mental health should be emphasized, with particular attention paid to its value in the prevention and intervention of social anxiety. Schools, communities, and mental health service agencies can incorporate group sports activities into mental health promotion programs. Through team sports such as basketball, soccer, and volleyball, they can increase interpersonal interaction and collaborative communication, helping participants enhance self-awareness and psychological resilience through exercise, and gradually alleviate social anxiety. At the same time, when organizing physical activities, emphasis should be placed on making them enjoyable, inclusive, and supportive, while avoiding an excessive focus on competition to prevent placing new psychological pressure on individuals who already experience social anxiety.

## Conclusion

1. Physical exercise, self-concept clarity, and psychological resilience are all correlated with social anxiety among college students in pairwise comparisons.
2. Physical exercise is positively correlated with self-concept clarity and psychological resilience among college students, and negatively correlated with social anxiety; self-concept clarity and psychological resilience are both negatively correlated with social anxiety.
3. Self-concept clarity and psychological resilience each act as independent mediators between physical exercise and social anxiety among college students.
4. Self-concept clarity and psychological resilience act as chain mediators between physical exercise and social anxiety among college students.

## Data Availability

The raw data supporting the conclusions of this article will be made available by the authors, without undue reservation.
